# Acute Airway Compromise Due to Suspected Fishbone Ingestion

**DOI:** 10.7759/cureus.86069

**Published:** 2025-06-15

**Authors:** Nirajan Nepal, Anna McKeone

**Affiliations:** 1 Department of Emergency Medicine, Madigan Army Medical Center, Tacoma, USA; 2 Department of Emergency Medicine, Providence St. Peter Hospital, Olympia, USA

**Keywords:** airway intubation, emergency airway management, fishbone ingestion, gastroenterology and endoscopy, laryngoscope, respiratory failure, swallowed foreign body, video laryngoscope

## Abstract

A 68-year-old woman presented to the emergency department (ED) with acute respiratory distress following suspected fishbone ingestion. The patient exhibited significant respiratory distress, and a video laryngoscope evaluation revealed bleeding and edema around the vallecula and arytenoid soft tissues, raising concerns for impending airway compromise. The patient was intubated and admitted to the intensive care unit (ICU) for further management. A soft tissue neck computed tomography scan revealed a fishbone lodged in the mid-esophagus, prompting a gastroenterology consultation for endoscopy. The endoscopy successfully removed the fishbone without complications. After the procedure, the patient remained stable and was monitored in the ICU. This case underscores the importance of early airway intervention in suspected foreign body ingestion and illustrates the role of prompt imaging and multidisciplinary management in ensuring favorable outcomes. Fortunately, the course following intervention was uneventful, and the patient was discharged with no further complications, highlighting the critical role of early intervention and careful management in preventing respiratory failure or other serious sequelae associated with foreign body ingestion.

## Introduction

Foreign body ingestion is a common emergency department (ED) presentation, particularly among pediatric and elderly populations. Ingestion of foreign objects is estimated to occur in approximately one in 3,000 to one in 5,000 individuals annually in the United States, with a higher prevalence among children under five years old and adults over 60 years old, who are more prone due to factors such as reduced protective airway reflexes, dental issues, and comorbidities [1,2]. While many ingested foreign bodies pass harmlessly through the gastrointestinal tract, certain objects, such as fishbones, pose a higher risk of complications due to their sharp structure and potential for mucosal injury. Fishbone ingestion is especially responsible for a significant number of cases of airway compromise, with studies reporting that foreign body aspiration accounts for 2%-4% of all ED visits for foreign body ingestion [3,4]. Fishbones, in particular, can lead to a spectrum of complications, including esophageal perforation, retropharyngeal abscess, mediastinitis, and airway compromise, with the risk increasing in elderly patients due to age-related changes in swallowing mechanics and airway reflexes [5].

When a foreign body becomes lodged in the upper aerodigestive tract, it can cause significant local inflammation, mucosal edema, and bleeding, which may progress to airway obstruction. The consequences of delayed recognition or intervention can be severe, including respiratory failure, sepsis, or death. In cases where patients present with respiratory distress, early airway assessment and intervention are critical to prevent deterioration. The use of laryngoscopy and fiberoptic airway evaluation in the emergency setting allows for direct visualization of the airway and can assist in determining the need for immediate airway management. Furthermore, imaging modalities such as X-rays and computed tomography (CT) scans can play a crucial role in locating the foreign body and guiding appropriate intervention.

## Case presentation

A 68-year-old woman presented to the ED with acute respiratory distress after consuming fish soup. She reported feeling a foreign body lodged in her throat and exhibited significant respiratory distress, an inability to speak, drooling, and minor oral bleeding. Due to her respiratory distress, her medical history was unobtainable. Her vital signs were as follows: blood pressure was 134/62 mmHg, heart rate was 51 beats/minute, temperature was 36.3°C, respiratory rate was 16 breaths/minute, and oxygen saturation was 98%.

The focused physical examination revealed that the patient was in distress, holding her anterior neck. There was saliva present with streaks of blood. A fishbone was not clearly visible in the oropharynx. While there was no stridor, the patient was experiencing significant difficulty managing secretions. There might be mild swelling of the lips and tongue, but it was uncertain if this was her baseline condition. Both lungs were clear to auscultation, with no wheezing detected.

Given the concern for airway compromise, an airway evaluation was performed using a fiberoptic laryngoscope under intravenous ketamine sedation. A dissociative dose of ketamine (0.5 mg/kg) was administered to provide sedation while maintaining airway reflexes and ensuring patient safety during the procedure.

Findings from the laryngoscopy included the following: bleeding from the right lower throat above the vallecula, edema around the vallecula and arytenoid soft tissues, no visible embedded fishbone, and normal vocal cords.

**Figure 1:**
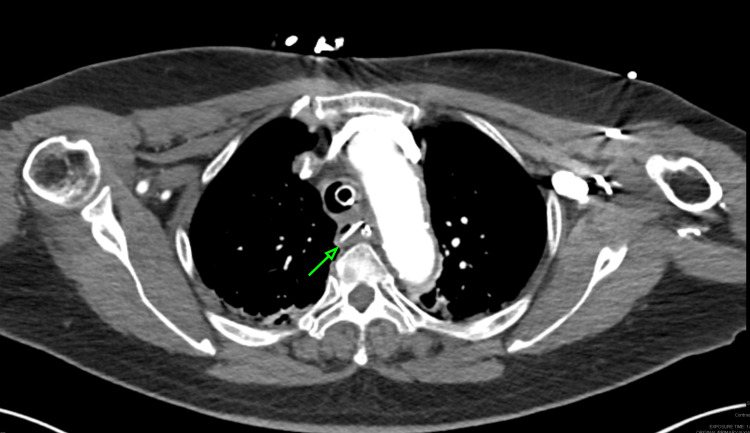
CT of the chest showing a well-positioned endotracheal tube within the trachea, along with a fishbone foreign body in the mid-esophagus. The green arrow identifies the fishbone CT: computed tomography

Progressive airway swelling and continued bleeding raised concerns for impending airway obstruction. The patient was promptly intubated using etomidate (0.3 mg/kg) for induction and rocuronium (1 mg/kg) as the paralytic agent with an initially difficult airway attempt. A chest X-ray, including lateral views obtained at the bedside, did not show a radiopaque foreign body. To assess for potential esophageal or tracheal involvement, a soft tissue neck and chest CT was ordered. The CT revealed a fishbone embedded in the mid-esophagus (Figure 1), with no signs of tracheal involvement or perforation.

**Figure 2:**
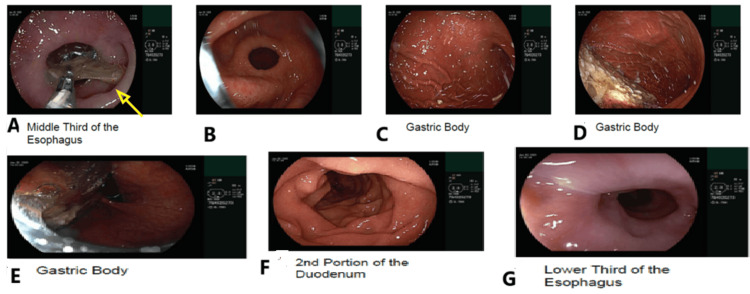
Emergent endoscopy visualizing a fishbone (top left, yellow arrow) in the middle third of the esophagus (A), followed by the successful retrieval of the fishbone (B), with subsequent imaging showing the other part of the esophagus without perforation or significant mucosal injury (C-G)

Following the CT scan, the gastroenterology service was consulted, and an endoscopy was performed, successfully removing the fishbone from the esophagus without complications (Figure 2). The patient remained stable after the procedure and was monitored in the intensive care unit for further management. The patient was successfully extubated the same day, passed the bedside swallow evaluation without any difficulties, and was discharged the following day without complications.

## Discussion

Foreign body ingestion, particularly fishbones, can indeed cause significant airway compromise. In cases of suspected ingestion with progressive symptoms, early airway assessment is crucial to prevent respiratory failure. Securing the airway is paramount in these cases, as delays in intervention can lead to severe complications such as hypoxia, aspiration, or even death. Early airway management techniques, including laryngoscopy and fiberoptic bronchoscopy, are critical to preventing further deterioration of the patient’s condition [1,2].

The use of a laryngoscope in the ED for foreign body evaluation is a critical procedure, particularly in cases of airway obstruction. Laryngoscopy allows for direct visualization of the airway and can facilitate the removal of foreign bodies, especially when they are located in the upper airway or hypopharynx. In the emergency setting, both direct and video laryngoscopes can be utilized. Direct laryngoscopy with a Macintosh or Miller blade is often employed for immediate visualization and removal of foreign bodies using tools such as Magill forceps [3]. Video laryngoscopes, such as the GlideScope (Verathon Inc., Bothell, WA), have been shown to provide enhanced visualization and can be particularly useful in difficult cases or when the foreign body is located in challenging anatomical positions [4]. Awake laryngoscopy is another technique that can be used in stable patients. This method allows for a controlled and deliberate examination of the airway, which can be beneficial in identifying and managing foreign bodies without the need for rapid sequence induction [5].

Fiberoptic airway evaluation is also an essential technique that allows for direct visualization of the airway and helps assess the extent of injury caused by a foreign body. According to White et al., fiberoptic bronchoscopy is a critical tool in the emergency management of airway foreign bodies, providing both diagnostic and therapeutic capabilities [4]. Early airway securement is critical in suspected foreign body aspiration with hemodynamic instability or respiratory distress. Techniques such as laryngoscopy, fiberoptic bronchoscopy, and even cricothyrotomy may be necessary to remove the obstructing foreign body and secure the airway [4]. Imaging plays a significant role in confirming the presence of a foreign body and guiding further intervention. Chest radiography and CT scans are commonly used for diagnosis. CT scans, in particular, have high sensitivity and specificity for detecting foreign bodies and can provide detailed information about their location and characteristics, which is crucial for planning the appropriate intervention [5].

In summary, the management of foreign body ingestion involves a combination of laryngoscopy, possible fiberoptic airway evaluation, early airway management, and imaging to confirm the presence and guide the removal of the foreign body. These steps are critical to prevent complications such as respiratory failure and to ensure optimal patient outcomes [4].

## Conclusions

This case highlights the critical importance of early airway assessment and intervention in suspected foreign body ingestion, particularly in patients at risk for airway compromise. Securing the airway early is crucial, with immediate intervention required in cases of respiratory distress or hemodynamic instability. The patient’s presentation necessitated prompt intubation to secure the airway. Although initial laryngoscopy did not reveal a visible foreign body, subsequent imaging confirmed a fishbone lodged in the esophagus, which was successfully removed via endoscopy without complications.

This case underscores the necessity of a multidisciplinary approach, incorporating emergency airway management, imaging, and endoscopic intervention, to ensure patient safety and prevent severe complications such as airway obstruction or perforation. Early recognition and timely intervention remain paramount in managing foreign body ingestion cases to prevent life-threatening respiratory failure.
